# A Bayesian Tensor Decomposition Method for Joint Estimation of Channel and Interference Parameters

**DOI:** 10.3390/s24165284

**Published:** 2024-08-15

**Authors:** Yuzhe Sun, Wei Wang, Yufan Wang, Yuanfeng He

**Affiliations:** School of Information Engineering, Chang’an University, Xi’an 710064, China; 2020024005@chd.edu.cn (Y.S.); 2021124100@chd.edu.cn (Y.W.); 2022124024@chd.edu.cn (Y.H.)

**Keywords:** robust Bayesian, automatic rank determination, interference estimation, tensor decomposition, channel estimation

## Abstract

Bayesian tensor decomposition has been widely applied in channel parameter estimations, particularly in cases with the presence of interference. However, the types of interference are not considered in Bayesian tensor decomposition, making it difficult to accurately estimate the interference parameters. In this paper, we present a robust tensor variational method using a CANDECOMP/PARAFAC (CP)-based additive interference model for multiple input–multiple output (MIMO) with orthogonal frequency division multiplexing (OFDM) systems. A more realistic interference model compared to traditional colored noise is considered in terms of co-channel interference (CCI) and front-end interference (FEI). In contrast to conventional algorithms that filter out interference, the proposed method jointly estimates the channel and interference parameters in the time–frequency domain. Simulation results validate the correctness of the proposed method by the evidence lower bound (ELBO) and reveal the fact that the proposed method outperforms traditional information-theoretic methods, tensor decomposition models, and robust model based on CP (RCP) in terms of estimation accuracy. Further, the interference parameter estimation technique has profound implications for anti-interference applications and dynamic spectrum allocation.

## 1. Introduction

In the past decade, OFDM with MIMO has become a widely adopted wireless transmission technique due to its ability to achieve high data rates [1,2] and enhance diversity gain and system capacity, particularly in scenarios with dynamic, time-varying, and frequency-selective channels [3,4]. In the context of big data processing, tensor-decomposition-based channel estimation methods [5,6] have attracted a significant amount of attention in MIMO-OFDM systems due to their high efficiency in processing large complex datasets with improved estimation accuracy for high-dimensional problems.

Tensor-decomposition-based channel estimation algorithms generally consist of two steps: the first step involves the estimation of rank, which corresponds to the number of multipath components, and the second step utilizes the obtained rank to estimate the multipath component parameters. It is widely acknowledged that determining the tensor rank is an NP-hard problem, as discussed in [7]. The predominant approaches to estimate the rank use information-theoretic methods, among which, the most popular methods are Akaike information criterion (AIC) and Bayesian information criterion (BIC), but these have drawbacks of oversimplification and overfitting [8]. Further, the minimum description length (MDL) [9] method demonstrates a significant reliance on prior knowledge and exhibits sensitivity. Based on the obtained rank, tensor decomposition can be applied to estimate the channel parameters. Based on the model of Tucker, M. Haardt [10] extended the high-order singular-value decomposition (HOSVD) to the estimation of channel parameters using the estimation of signal parameters via rotational invariance techniques (ESPRIT). Further application has been expanded to 5G localization mapping as described in [11]. Based on the model of CP, an enhanced approach was proposed in [12] to address the downlink channel estimation problem in MIMO-OFDM systems with large antenna arrays. Further enhancement was conducted in [13], where a tensor-space-assisted estimation scheme was proposed by exploiting the Vandermonde structure of the factor matrix. In addition, based on the two aforementioned models, the sequential unfolding singular-value decomposition (SUSVD) was proposed in [14] by utilizing a distinctive hierarchical tree structure to obtain orthogonal factor matrices: also called the PARATREE method.

Due to the presence of interference, the performance of traditional tensor-decomposition-based channel estimation methods is severely degraded, as the actual channel interference cannot be simply modeled as colored noise. The degradation of rank estimation performance significantly reduces the performance of channel parameter estimation. This is particularly true in MIMO systems, where interference exhibits high correlation. Interference primarily arises from imperfect designs in MIMO-OFDM systems and front-end circuits, leading to signal distortion, including harmonic distortion, intermodulation distortion, and phase distortion, as demonstrated by radio frequency FEI [15,16]. Additionally, frequency reuse and bandwidth congestion may also result in CCI [17,18,19]. When the same frequency bands are allocated to multiple transmitters, signal overlap and degradation usually occur. Traditional methods for addressing interference have relied on additional hardware and post-processing algorithms to filter out interference. However, emerging efficient spectrum allocation technologies based on spectrum sensing and interference identification have significant research implications [20,21].

Based on a non-Gaussian and non-stationary interference model, tensor decomposition can be employed to reduce the dimensionality of multi-dimensional data. Qibin Zhao proposed a tensor-based variational Bayesian approach in [22] for channel estimation by eliminating interference in the channel matrix and utilizing the spatial coupling relationships of partially received tensors. References [23,24] proposed to use threshold-based interference exclusion methods for low-rank approximation and channel parameter estimation with incomplete data. In [25], a multiplicative Gamma process (MGP) was used to reduce the complexity and enhance the speed of automatic rank determination (ARD). Similarly, the use of a generalized hyperbolic (GH) distribution can achieve more flexible sparsity awareness [26]. It is worth mentioning that variational methods with incomplete observations still suffer from information entropy loss with the presence of interference.

Therefore, it is essential to incorporate channel information, including the additive interference structure. Traditional methods like adaptive filtering [27], prior-knowledge-based MIMO systems [28], and radio frequency (RF) front-end feedback networks [29] focus on removing rather than estimating the interference. Moreover, these conventional approaches have drawbacks of high complexity and costs. An additive RCP [30] was proposed by inferring interference terms for each pixel in image processing to enhance the precision of image processing, which was expanded to channel parameter estimation in [31]. However, so far, the actual types of interference in the tensor have not yet been thoroughly considered in the model, which makes accurate interference estimation difficult.

In this paper, we propose an RCP based on alternate prior hypothesis (APH) for channel estimation in MIMO-OFDM systems, hereinafter referred to as RCP-APH. We first separate the interference tensor space and then construct spatial correlations through actual interferences, either FEI or CCI. Consequently, we perform variational iterations in the separated tensor space by alternately modifying the interference prior hypotheses conditions. The main contributions of this paper are summarized as follows:

- We adopt an additive interference model for which the parameters are jointly estimated with channel parameters rather than mitigating the interference. As such, it has profound implications in anti-interference applications and dynamic spectrum allocation.
- We propose to jointly estimate the channel and interference parameters without increasing the complexity and degrading the estimation performance. The proposed method enables simultaneously estimating the number of paths and the channel and interference parameters in MIMO-OFDM systems.

The structure of this paper is as follows. Section 2 describes the preliminaries and basic concepts. Section 3 presents the MIMO-OFDM system model. In Section 4, we propose the RCP-APH algorithm, and Section 5 shows the experimental analysis of the proposed algorithm. Finally, Section 6 summarizes this paper.

## 2. Preliminaries and Notations

In this paper, we introduce the term “mode”, denoted by *n*, to represent the order of a tensor, which is also referred to as the dimension in various disciplines. An *N*-th order complex tensor is represented using calligraphic letters, as illustrated by $X\in C^{I_{1}\times I_{2}\times \cdots \times I_{N}}$, for which the $\left(i_{1},i_{2},\cdots ,i_{N}\right)$ entry is denoted by $X_{i_{1},i_{2},\cdots ,i_{N}}$, $i_{n}=1,2,\cdots ,I_{n}$, n=1,2,⋯,N. Furthermore, the unfolding of the tensor X with respect to the *n*-th mode is represented by $X_{\left(n\right)}$ in accordance with [10].

Tensors are sliced along different dimensions to form a sub-tensor, which is also known as tensor slicing. Slices of a three-dimensional tensor are represented as matrices and are denoted by uppercase bold letters. A set of data along a specific dimension of the tensor is referred to as a fiber and is represented in vector form and denoted by lowercase bold letters. Therefore, in the context of a three-dimensional tensor, the relationship between a slice and a fiber is expressed as $X_{:,:,i_{3}}=X={\left[x_{1},\ldots ,x_{i_{1}},\ldots ,x_{I_{1}}\right]}^{T}$, where the row vector of the slice is represented as $X_{i_{1},:,i_{3}}=x_{i_{1}}={\left[x_{i_{1},1},\cdots ,x_{i_{1},i_{2}},\cdots ,x_{i_{1},I_{2}}\right]}^{T}$. Throughout this paper, We use the symbols ∗, *T*, *H*, −1, $\tilde{▪}$, $\left\{▪\right\}$, and ${∥▪∥}_{F}$ to denote the conjugate, transposition, Hermitian transposition, matrix inversion, estimated value, and set of the same and Frobenius norm operations, respectively.

For multilinear mathematical operations, the complex inner product of vectors is defined by $\langle x_{i_{1}}^{\left(1\right)},x_{i_{2}}^{\left(2\right)},\cdots ,x_{i_{N}}^{\left(N\right)}\rangle =\sum_{r}\prod_{n}x_{i_{n},r}^{{\left(n\right)}^{*}}=x_{i_{n}}^{(n)H}\left(\underset{k\neq n}{⊛}\;x_{i_{k}}^{(k)*}\right)$. The Hadamard product is performed in an entrywise way between two items of the same size, such as $A\in C^{I\times J}$ and $B\in C^{I\times J}$ matrices, and the result is $A⊛B\in C^{I\times J}$. The Kronecker product of matrices $A\in C^{I\times J}$ and $B\in C^{K\times L}$ is a matrix of size IK×JL, denoted by A⊗B. The Khatri–Rao product of matrices, $A\in C^{I\times K}$ and $B\in C^{J\times K}$, is $A⊙B\in C^{IJ\times K}$, which is defined by a columnwise Kronecker product. Without loss of generality, the Hadamard product and Khatri–Rao product of a set of matrices, except the *n*-th matrix, can be simply denoted by

(1) $$\begin{array}{c} \underset{k\neq n}{⊛}\;A^{\left(k\right)}=A^{\left(N\right)}⊛\cdots ⊛A^{\left(n+1\right)}⊛A^{\left(n-1\right)}\cdots ⊛A^{\left(1\right)},\\ \underset{k\neq n}{⊙}\;A^{\left(k\right)}=A^{\left(N\right)}⊙\cdots ⊙A^{\left(n+1\right)}⊙A^{\left(n-1\right)}\cdots ⊙A^{\left(1\right)}. \end{array}$$

## 3. MIMO-OFDM System Model

**Figure 1:**
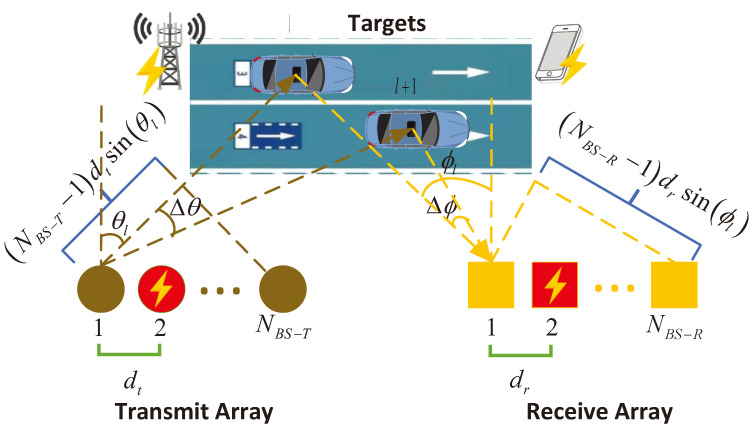
A typical traffic scenario.

We consider a typical traffic multipath scenario with the presence of interference as depicted in Figure 1. The transmit and receive array consist of $N_{BS-T}$ and $N_{BS-R}$ antennas with equidistant spacing of $d_{t}$ and $d_{r}$, respectively. The linear arrays at both ends form a MIMO system designed to estimate channel parameters, including the angle of departure (AoD) θ, the angle of arrival (AoA) ϕ, the delay τ, and the complex amplitude α. It can be observed that the parameter set for the *l*-th multipath is $\left\{\theta_{l},\phi_{l},\tau_{l},\alpha_{l}\right\}$, and the (l+1)-th path has angular differences in the transmission angle Δθ and arrival angle Δϕ compared to the former path. In this paper, we use an OFDM signal with a bandwidth of *B* and modulated by *K* subcarriers for transmission. For convenience, the *K* subcarriers with a spacing of B/K are all used to transmit periodic known training pilots. The periodicity of the signal ensures that the end of each OFDM symbol naturally connects with the beginning of the next symbol. We assume that the signal has been detected and synchronized, where the whole piece of hte signal symbol is recovered for channel estimation [32]. And there are *L* paths in the propagation channel. At the receiver, by utilizing the orthogonality of transmission symbols and stacking the channel matrices of *K* frequency points, we can get the channel tensor $H\in C^{N_{BS-R}\times N_{BS-T}\times K}$ in the form of CP factorization as follows:

(2) $$\begin{array}{c} \begin{array}{c} H=\sum_{l=1}^{L}a_{\mathrm{BS}-R}\left(\phi_{l}\right)\circ a_{\mathrm{BS}-T}\left(\theta_{l}\right)\circ \left(\alpha_{l}g\left(\tau_{l}\right)\right)=\left[\left[A^{\left(1\right)},A^{\left(2\right)},A^{\left(3\right)}\right]\right] \end{array},\\ A^{\left(1\right)}≜\left[a_{\mathrm{BS}-R}\left(\phi_{1}\right),a_{\mathrm{BS}-R}\left(\phi_{2}\right),\cdots ,a_{\mathrm{BS}-R}\left(\phi_{L}\right)\right],\\ A^{\left(2\right)}≜\left[a_{\mathrm{BS}-T}\left(\theta_{1}\right),a_{\mathrm{BS}-T}\left(\theta_{2}\right),\cdots ,a_{\mathrm{BS}-T}\left(\theta_{L}\right)\right],\\ A^{\left(3\right)}≜\left[\alpha_{1}g\left(\tau_{1}\right),\alpha_{2}g\left(\tau_{2}\right),\cdots ,\alpha_{L}g\left(\tau_{L}\right)\right], \end{array}$$

where “∘” indicates the outer product, factor matrices ${\left\{A^{\left(n\right)}\right\}}_{n=1,2,3}$ are composed of the corresponding antenna array response, $g\left(\tau_{l}\right)≜[\exp\left(-j2\pi \tau_{l}B\left(1/K\right)\right).,$ $\exp(-j2\pi \tau_{l}B\left(2/K\right)),$ ⋯, ${.\exp(-j2\pi \tau_{l}B)]}^{T}$, $a_{\mathrm{BS}-R}\left(\phi_{l}\right)=[\begin{array}{cc} 1 & e^{j\mu (\phi_{l})} \end{array}.$ $.\cdots \;e^{j\left(N_{BS-R}-1\right)\mu \left(\phi_{l}\right)}{]}^{T}$, and $a_{\mathrm{BS}-T}\left(\theta_{l}\right)=[\begin{array}{cc} 1 & e^{j\mu (\theta_{l})} \end{array}.$ $.\cdots \;e^{j\left(N_{BS-T}-1\right)\mu \left(\theta_{l}\right)}{]}^{T}$. At the same time, the phases of these are respectively represented by $\mu \left(\phi_{l}\right)=\left(2\pi /\lambda_{c}\right)d_{r}\sin\phi_{l}$ and $\mu \left(\theta_{l}\right)=\left(2\pi /\lambda_{c}\right)d_{t}\sin\theta_{l}$, where $\lambda_{c}$ is the signal wavelength.

**Figure 2:**
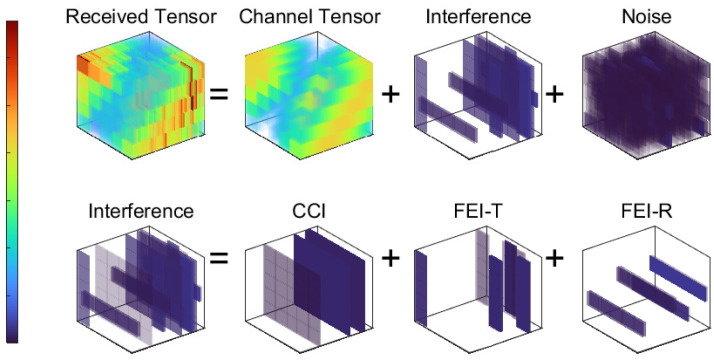
The power composition of the received tensor.

We assume additive interference, as seen in Figure 2. The frequency power composition of the received tensor is composed as Y=H+S+W, where H, S, and W represent a channel tensor with the channel information, channel interference, and the noise tensor, respectively, which all follow an independent and identical distribution (i.i.d.). It is essential to note that the yellow lightning inside the red circle in Figure 1 indicates the FEI of the transmitter antenna, denoted as $S^{FEI-T}$. Similarly, the yellow lightning inside the red square represents the FEI of the receiver antenna, represented by $S^{FEI-R}$. Following that, the yellow lightning appearing on both sides of the road indicates CCI generated by other electronic devices and neighboring cells, referred to as $S^{CCI}$. Therefore, the interference tensor of FEI-R is made of a row fiber with a size of $1\times N_{BS-R}$, indicating this FEI-R from a particular receiving antenna to all transmitting sub-channels. In the same way, the interference tensor FEI-T is made of a column fiber with a size of $N_{BS-T}\times 1$, indicating this FEI-T is from a particular transmitting antenna and affects all receiving sub-channels. In addition, the interference is assumed to occur at any possible spectrum location and to have an arbitrary amplitude and phase. Section 5.4 describes the characteristics of the proposed algorithm for different interference bandwidths.

## 4. Bayesian Tensor Factorization

Diverging from the traditional RCP algorithm [30], this paper involves a strong correlation assumption about the prior information about interference. Spatially, this correlation is established on the entire slice and on the fibers in both the horizontal and vertical directions. Under the condition of maximizing the evidence, different interferences are alternately estimated.

### 4.1. Alternate Prior Hypotheses

To alleviate the complexity in the description, a third-order CP generative model is employed. As previously mentioned, the full-set representation of subscripts is denoted as $\Omega =\{i_{1},i_{2},i_{3}\}$, where $i_{n}\in \left[1,\cdots ,I_{n}\right]$ and n=1,2,3. It corresponds to the actual configuration, as $I_{1}=N_{BS-R},I_{2}=N_{BS-T}$ and $I_{3}=K$. In order to achieve RCP within a probabilistic framework, an observation model is introduced:

(3) $$\begin{array}{c} p\left(Y_{\Omega }|{\left\{A^{\left(n\right)}\right\}}_{n=1}^{3}S_{\Omega },v\right)=\prod_{i_{1},i_{2},i_{3}}CN\left(Y_{i_{1},i_{2},i_{3}}|\langle a_{i_{1}}^{\left(1\right)},a_{i_{2}}^{\left(2\right)},a_{i_{3}}^{\left(3\right)}\rangle +S_{i_{1},i_{2},i_{3}},v^{-1}\right), \end{array}$$

where ν denotes the noise precision, $S_{\Omega }$ represents all items of interference, and one interference term is denoted as $S_{i_{1},i_{2},i_{3}}$. Each vector $a_{i_{n}}^{\left(n\right)}$ influences a sub-tensor with index $i_{n}$ under mode-n. The generalized inner product $\langle a_{i_{1}}^{\left(1\right)},a_{i_{2}}^{\left(1\right)},a_{i_{3}}^{\left(3\right)}\rangle$ of the three latent vectors enables us to capture multilinear interactions reflecting the intrinsic structural characteristics of the tensor data. But this “inner product” complicates the learning process of the model. Therefore, an attempt is made to minimize the dimensionality of the latent space by inducing sparsity in the columns of the factor matrices:

(4) $$\begin{array}{c} p\left(A^{\left(n\right)}|\lambda \right)\;=\prod_{i_{n}}CN\left(\left.a_{i_{n}}^{\left(n\right)}\right|0,\Lambda^{-1}\right),\\ p\left(\lambda \right)=\prod_{l=1}^{L}\mathrm{Ga}\left(\left.\lambda_{l}\right|c_{0},d_{0}\right), \end{array}$$

where Λ=diag(λ) represents the inverse covariance matrix, and $\lambda =\left[\lambda_{1},\lambda_{2},\cdots ,\lambda_{L}\right]$ is shared by the factor matrices across all modes. Due to the uncorrelation of the channel multipath, these hyperpriors for λ assume an i.i.d. hypothesis. The Gamma distribution is denoted as $\mathrm{Ga}\left(x\left|m,n\right.\right)=n^{m}x^{m-1}e^{-nx}/\Gamma \left(m\right)$, where $\Gamma \left(m\right)$ is the Gamma function. Furthermore, considering the zero-mean complex Gaussian distribution characteristics of the actual channel’s amplitudes, we can draw a conclusion for the ARD: that is, when a certain path $\lambda_{l}$ is sufficiently large, the corresponding *l*-th column of the latent factor matrix tends to zero, thereby removing the corresponding redundant path.

For the convenience of subsequent discussions, a category of interferences is denoted as $S^{Tp}$, as previously discussed in Figure 2. An individual interference from this category is represented as $S_{Ind\left(Tp\right)}^{Tp}$, and its specific position in the time–frequency domain is determined by the subscript index $Ind\left(Tp\right)$, including $Ind\left(CCI\right)=\left\{i_{3}\right\}$, $Ind\left(FEI-R\right)=\left\{i_{1},i_{3}\right\}$, and $Ind\left(FEI-T\right)=\left\{i_{2},i_{3}\right\}$. The complete set of interference terms for a certain category is represented as $S_{\Omega }^{Tp}$, with an individual interference term denoted as $S_{i_{1},i_{2},i_{3}}^{Tp}$. Thus, taking the condition of mutual independence between different interferences into account, the following interference prior assumptions are made:

(5) $$\begin{array}{c} p\left(\left.S^{Tp}\right|\gamma^{Tp}\right)=\prod_{Ind(Tp)}CN\left(\left.S_{Ind(Tp)}^{Tp}\right|0,1/\gamma_{Ind(Tp)}^{Tp}\;\right),\\ p\left(\gamma^{Tp}\right)=\prod_{Ind(Tp)}\mathrm{Ga}\left(\left.\gamma_{Ind(Tp)}^{Tp}\right|a_{0}^{Tp},b_{0}^{Tp}\right), \end{array}$$

where the different types of Tp correspond to the different hyperparameters $\gamma^{Tp}$. Moreover, according to the different resolution priors described in Section 3, the relationships between the interference and interference terms are given by $1_{I_{1}\times I_{2}}S_{i_{3}}^{CCI}=S_{:,:,i_{3}}^{CCI}$, $1_{I_{2}}^{T}S_{i_{1},i_{3}}^{FEI-R}=S_{i_{1},:,i_{3}}^{FEI-R}$, and $1_{I_{1}}S_{i_{2},i_{3}}^{FEI-T}=S_{:,i_{2},i_{3}}^{FEI-T}$. This also indicates that the interference cannot be simply considered to be a Gaussian distribution, nor can it be simply modeled as colored noise. Finally, a hyperprior is placed on the noise precision of the environment:

(6) $$p\left(\nu \right)=\mathrm{Ga}\left(\left.\nu \right|e_{0},f_{0}\right).$$

**Figure 3:**
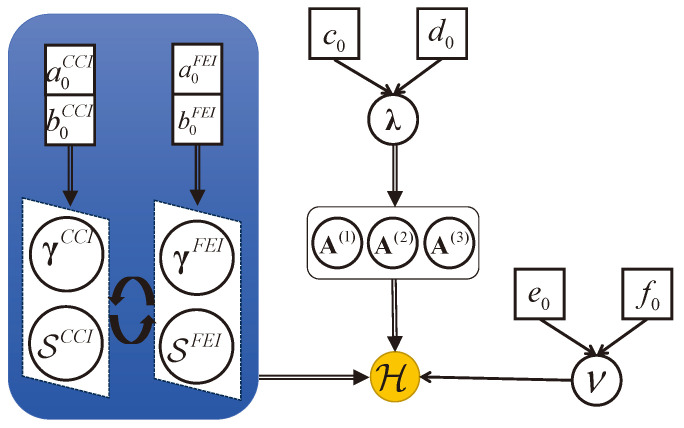
Probabilistic graphical model.

All the mentioned prior assumptions have been assumed within the probabilistic graphical model, as illustrated in Figure 3. In this figure, white circles and squares represent hidden random variables and hyperparameters, respectively, while yellow circles denote the observed tensor. In the blue region, it is clear that this variational method is implemented through alternating iterations between two priors, including CCI and FEI.

### 4.2. Variational Bayesian Inference

For simplicity, all factor matrices and hyperparameters are integrated into the parameter set $\Theta =\{A^{\left(1\right)},A^{\left(2\right)},A^{\left(3\right)},\lambda ,$ $S^{\Xi },\gamma^{\Xi },\nu \}$, and $\Xi =\left\{CCI,FEI-R,FEI-T\right\}$. Consequently, with different types of interference, the likelihood function is obtained as follows:

(7) $$\begin{array}{cc} p\left(Y_{\Omega }-{\tilde{S}}_{\Omega }^{\backslash Tp},\Theta \right) & =p\left(Y_{\Omega }-{\tilde{S}}_{\Omega }^{\backslash Tp}|{\left\{A^{\left(n\right)}\right\}}_{n=1}^{3},S^{Tp},\nu \right)\prod_{n=1}^{3}p\left(A^{\left(n\right)}|\lambda \right)\\ \; & \cdot p\left(S^{Tp}|\gamma^{Tp}\right)p\left(\lambda \right)p\left(\gamma^{Tp}\right)p\left(\nu \right), \end{array}$$

where the symbol “∖” denotes the complement of the set—for example, when Tp=CCI, $\backslash Tp=\left\{FEI-R,FEI-T\right\}$—and ${\tilde{S}}^{\backslash Tp}$ represents the estimated values of the remaining two types of interferences, such as ${\tilde{S}}^{\backslash CCI}={\tilde{S}}^{FEI-R}+{\tilde{S}}^{FEI-T}$. The variational approach involves approximating the posterior distribution $p\left(\Theta |Y_{\Omega }-{\tilde{S}}_{\Omega }^{\backslash Tp}\right)$ with the distribution of q(Θ), and the relationship is as follows:

(8) $$\begin{array}{c} \ln p\left(Y_{\Omega }-{\tilde{S}}_{\Omega }^{\backslash Tp}\right)=\mathrm{KL}\left(q\left(\Theta \right)∥p\left(\Theta |Y_{\Omega }-{\tilde{S}}_{\Omega }^{\backslash Tp}\right)\right)+L\left(q,Tp\right). \end{array}$$

In the above equation, the evidence of $p\left(Y_{\Omega }-{\tilde{S}}_{\Omega }^{\backslash Tp}\right)$ remains a constant, so maximizing the ELBO of parameter L(q,Tp) will inevitably minimize the Kullback–Leibler (KL) divergence, thereby completing the inference for the posterior distribution. In this process, given the uncorrelated characteristics of actual parameters, the uniform field theory is employed as follows:

(9) $$q\left(\Theta \right)=\prod_{n=1}^{3}q\left(A^{\left(n\right)}\right)q\left(S^{Tp}\right)q\left(\lambda \right)q\left(\gamma^{Tp}\right)q\left(\nu \right).$$

Finally, by computing the expectation of the log-likelihood function $\ln p\left(Y_{\Omega }-{\tilde{S}}_{\Omega }^{\backslash Tp},\Theta \right)$ under the posterior distribution $q\left(\Theta /\Theta_{j}\right)$ of the remaining parameters, precise posterior inference for this parameter $\Theta_{j}$ is obtained as:

(10) $$\ln q_{j}\left(\Theta_{j}\right)=E_{q_{\left(\Theta \backslash \Theta_{j}\right)}}\left[\ln p\left(Y_{\Omega }-{\tilde{S}}_{\Omega }^{\backslash Tp},\Theta \right)\right]+\mathrm{const}.$$

#### 4.2.1. Posterior Distribution of Factor Matrices $A^{\left(n\right)}$

By using Equation (8), after performing the posterior expectation on all unknown latent variables and hyperparameters, except the n-mode matrix $A^{\left(n\right)}$, the distribution follows a complex Gaussian distribution $q_{n}\left(A^{\left(n\right)}\right)=CN\left(\left.A^{\left(n\right)}\right|{\tilde{A}}^{\left(n\right)},V^{\left(n\right)}\right)$, for which the mean and variance are

(11) $$\begin{array}{cc} {\tilde{A}}^{\left(n\right)} & =\left({\left[Y_{\Omega }-{\tilde{S}}_{\Omega }^{\backslash Tp}\right]}_{\left(n\right)}-E_{q}\left[{\left(S_{\Omega }^{Tp}\right)}_{\left(n\right)}\right]\right)\cdot E_{q}\left[A^{(\backslash n)*}\right]V^{\left(n\right)}E_{q}\left[\nu \right],\\ V^{\left(n\right)} & ={\left\{E_{q}{\left[A^{(\backslash n)H}A^{\left(\backslash n\right)}\right]}^{T}\cdot E_{q}\left[\nu \right]+E_{q}\left[\Lambda \right]\right\}}^{-1}, \end{array}$$

where $E_{q}\left[\cdot \right]$ represents the posterior expectation, $A^{\left(\backslash n\right)}=\underset{k\neq n}{⊙}\;A^{\left(k\right)}$, and $E_{q}\left[\Lambda \right]=\mathrm{diag}\left(E_{q}\left[\lambda \right]\right)$ $=\tilde{\Lambda }$. It should be noted that the derivation in this paper utilizes the uncorrelated characteristics between factor matrices from different modes as well as the uncorrelated characteristics among different row vectors of the same factor matrix. The above assumption aligns perfectly with the prior assumptions of the actual channel tensor.

#### 4.2.2. Posterior Distribution of Hyperparameters λ

As assumed in Equation (4), we have: $q_{\lambda }\left(\lambda \right)=\prod_{l=1}^{L}\mathrm{Ga}\left(\left.\lambda_{l}\right|c_{M}^{l},d_{M}^{l}\right)$, where $c_{M}^{l},d_{M}^{l}$ denote the posterior parameters learned from observations and can be updated by:

(12) $$\begin{array}{c} c_{M}=\left(c_{0}+\sum_{n=1}^{3}I_{n}\right)1_{L},\\ d_{M}=d_{0}1_{L}+\sum_{n=1}^{3}\left\{diag\left({\tilde{A}}^{(n)H}{\tilde{A}}^{\left(n\right)}+I_{n}V^{\left(n\right)}\right)\right\}, \end{array}$$

where vector $c_{M}=\left[c_{M}^{1},c_{M}^{2},\cdots ,c_{M}^{L}\right]$, and $d_{M}=[d_{M}^{1},d_{M}^{2},$ $\cdots ,d_{M}^{L}]$. With regard to the prior knowledge, we know the posterior $E_{q}\left[\lambda \right]=[c_{M}^{1}/d_{M}^{1},\cdots ,$ $c_{M}^{L}/d_{M}^{L}]$, which directly determines whether a certain path should be eliminated. Because the mapping relationship between interference disrupts the Gaussian prior distribution at the lattice level, it reduces the algorithm’s generalization capability and decreases the speed of calculating the variational expectation. Therefore, in this paper, we set a multiplicative threshold η to perform principal component analysis (PCA) according to the maximum and minimum values in $E_{q}\left[\lambda \right]$. This method significantly improves the speed of convergence, as shown in Section 5.2.

#### 4.2.3. Posterior Distribution of Hyperparameters S

In practical situations, different interferences are uncorrelated, and interferences from the same type at different frequency points are also uncorrelated. Therefore, we get the posterior distribution for $q\left(S^{Tp}\right)=\prod_{Ind(Tp)}CN\left(S_{Ind(Tp)}^{Tp}|{\tilde{S}}_{Ind(Tp)}^{Tp},{\left(\sigma_{Ind(Tp)}^{TP}\right)}^{2}\right)$ as:

(13) $$\begin{array}{cc} \; & {\tilde{S}}_{Ind\left(Tp\right)}^{Tp}=\frac{{\left(\sigma_{Ind\left(Tp\right)}^{Tp}\right)}^{2}E_{q}\left[\nu \right]}{Cst\left[\backslash Ind\left(Tp\right)\right]}\cdot \sum_{\backslash Ind\left(Tp\right)}\left\{Y_{i_{1},i_{2},i_{3}}-{\tilde{S}}_{i_{1},i_{2},i_{3}}^{\backslash Tp}-E_{q}\left[\langle a_{i_{1}}^{\left(1\right)},a_{i_{2}}^{\left(2\right)},a_{i_{3}}^{\left(3\right)}\rangle \right]\right\},\\ \; & {\left(\sigma_{Ind\left(Tp\right)}^{Tp}\right)}^{2}={\left(E_{q}\left[\nu \right]+E_{q}\left[\gamma_{Ind\left(Tp\right)}^{Tp}\right]\right)}^{-1}. \end{array}$$

**Figure 4:**
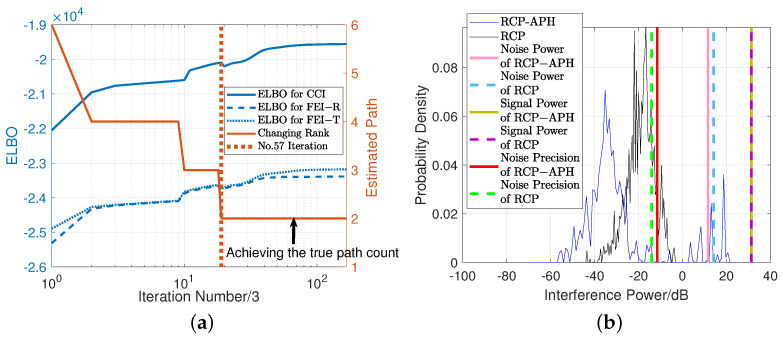
(**a**) The changes in the number of paths and the three variations of ELBO for RCP-APH. (**b**) The probability density function (PDF) of the interference item power distribution and other estimated parameters for RCP and RCP-APH.

In the above equation, when Tp is equal to CCI, the remaining terms’ operation is $\backslash Ind\left(CCI\right)=\left\{i_{1},i_{2}\right\}$. $Cst\left[\cdot \right]$ denotes the multiplication of the maximum index, resulting in $Cst\left[i_{1},i_{2}\right]=I_{1}I_{2}$. It is evident that in the posterior estimation of each interference type, the global information $E_{q}\left[\langle a_{i_{1}}^{\left(1\right)},a_{i_{2}}^{\left(2\right)},a_{i_{3}}^{\left(3\right)}\rangle \right]$ of the channel must be utilized, while the interference variance is determined by environmental Gaussian noise $E_{q}\left[\nu \right]$. Therefore, accurately estimating the noise is a crucial prerequisite for precisely assessing interference, as illustrated in Figure 4b.

#### 4.2.4. Posterior Distribution of Hyperparameters γ

In the prior assumption of Equation (5), the estimation of interference precision directly dictates the presence of interference, so we need to set an appropriate interference power threshold (IPTH), as discussed in Section 5.2. The posterior distribution, $q\left(\gamma^{Tp}\right)=\prod_{Ind(Tp)}Ga\left(\left.\gamma_{Ind(Tp)}^{Tp}\right|a_{M}^{\gamma_{Ind(Tp)}},b_{M}^{\gamma_{Ind(Tp)}}\right)$, is represented by the following equation:

(14) $$\begin{array}{c} a_{M}^{\gamma_{Ind(Tp)}}=Cst\left(\backslash Ind\left(Tp\right)\right)+a_{0}^{Tp},\\ b_{M}^{\gamma_{Ind(Tp)}}=b_{0}^{Tp}+\sum_{\backslash Ind(Tp)}E_{q}\left[{\left|S_{i_{1},i_{2},i_{3}}^{Tp}\right|}^{2}\right], \end{array}$$

where the prior hyperparameter $b_{0}^{Tp}$ is assumed for a specific type of interference, and the posterior hyperparameter $b_{M}^{\gamma_{Ind(Tp)}}$ varies with different indices $Ind\left(Tp\right)$.

#### 4.2.5. Posterior Distribution of Hyperparameters ν

The inference of noise precision is achieved through three factor matrices and observed data. Its posterior follows a Gamma distribution $q\left(\nu \right)=\mathrm{Ga}\left(\nu |e_{M},f_{M}\right)$, determined by the following:

(15) $$\begin{array}{cc} e_{M} & =\prod_{n=1}^{3}I_{n}+e_{0},\\ f_{M} & =f_{0}+E_{q\left(\Theta \backslash \nu \right)}\left[{∥Y_{\Omega }-{\tilde{S}}_{\Omega }^{\backslash Tp}-\left[\;\left[A^{\left(1\right)},A^{\left(2\right)},A^{\left(3\right)}\right]\;\right]∥}_{F}^{2}-S_{\Omega }^{Tp}\right], \end{array}$$

where the expectation operation is expressed in Equation (16), where the $i_{n}$-th row of $F^{\left(n\right)}$ is denoted by $f_{i_{n}}^{\left(n\right)}=vec\left[E_{q}{\left(a_{i_{n}}^{\left(n\right)}a_{i_{n}}^{(n)H}\right)}^{T}\right]$.

(16) EqΘ\νYΩ−S˜Ω\Tp−A(1),A(2),A(3)F2−SΩTp=YΩ−S˜Ω\TpF2−2RevecHYΩ−S˜Ω\TpvecS˜ΩTp+1∏nTIn⊙nF(n)1L2−2RevecHYΩ−S˜Ω\TpvecA˜(1),A˜(2),A˜(3)+EqSΩTpF2+2RevecHA˜(1),A˜(2),A˜(3)vecS˜ΩTp.

### 4.3. Evidence Lower Bound

From Equation (8), it can be observed that the algorithm conducts variational inference from three dimensions. Naturally, under the accurate elimination of redundant paths, the evidence lower bound (ELBO) undergoes a monotonically non-decreasing iterative process. The concept of maximizing ELBO involves the posterior expectation of the joint distribution and the entropy of the posterior distribution. The derivation of ELBO can be expressed as Equation (17) (see Appendix A for details), where we divide different dimensions with Tp, allowing for the validation of each dimension separately, as shown in Figure 4a.

(17) $$\begin{array}{cc} \; & L\left(q,Tp\right)=E_{q(\Theta )}\left[\ln p\left(Y-{\tilde{S}}^{\backslash Tp},\Theta \right)\right]+H\left(q\left(\Theta \right)\right)\\ \; & =-\frac{e_{M}}{f_{M}}E_{q}\left[{∥Y_{\Omega }-{\tilde{S}}_{\Omega }^{\backslash Tp}-\left[\;\left[A^{\left(1\right)},A^{\left(2\right)},A^{\left(3\right)}\right]\;\right]-S_{\Omega }^{Tp}∥}_{F}^{2}\right]-\mathrm{Tr}\left\{\tilde{\Lambda }\sum_{n}\left({\tilde{A}}^{(n)H}{\tilde{A}}^{\left(n\right)}+I_{n}V^{\left(n\right)}\right)\right\}\\ \; & +\sum_{n}I_{n}\left|V^{\left(n\right)}\right|+\sum_{l}\left\{\ln\Gamma \left(c_{M}^{l}\right)+c_{M}^{l}\left(1-\ln d_{M}^{l}-\frac{d_{0}^{l}}{d_{M}^{l}}\right)\right\}+e_{M}\left(1-\frac{f_{0}}{f_{M}}-\ln f_{M}\right)\\ \; & +\sum_{Ind(Tp)}\left\{\ln\Gamma \left(a_{M}^{\gamma_{Ind(Tp)}}\right)+a_{M}^{\gamma_{Ind(Tp)}}\left(1-\frac{b_{0}^{Tp}}{b_{M}^{\gamma_{Ind(Tp)}}}-\ln b_{M}^{\gamma_{Ind(Tp)}}\right)\right\}+\ln\Gamma \left(e_{M}\right)\\ \; & -\sum_{Ind(Tp)}\left(\sum_{\backslash Ind(Tp)}\left[\frac{a_{M}^{\gamma_{Ind(Tp)}}}{b_{M}^{\gamma_{Ind(Tp)}}}\left[{\left(\sigma_{i_{1},i_{2},i_{3}}^{Tp}\right)}^{2}+{\left|{\tilde{S}}_{i_{1},i_{2},i_{3}}^{Tp}\right|}^{2}\right]-\ln{\left(\sigma_{i_{1},i_{2},i_{3}}^{Tp}\right)}^{2}\right]\right)\\ \; & +Cst\left(Ind(Tp)\right)\left(a_{0}^{Tp}\ln b_{0}^{Tp}-\ln\Gamma \left(a_{0}^{Tp}\right)\right)+\mathrm{const} \end{array}.$$

### 4.4. Computational Complexity

The time complexity of the three factor matrices in Equation (11) is $O(3L^{3}+3ML+\sum_{n}I_{n}L^{2})$, where the total size of the observed data is $M=\prod_{n}I_{n}$, and *L* represents the number of multipaths and the model complexity. The computational cost for λ in Equation (12) is $O(\sum_{n}I_{n}L^{2})$. And similarly, the computational cost for ν is $O(ML^{2})$. So far, for the calculation of the above parameters, the proposed method has the same computational complexity as the traditional RCP algorithm. Furthermore, since this algorithm performs iterations at different resolutions for the classified interference $S^{Tp}$, the computational complexities for each iteration in terms of CCI, FEI-R, and FEI-T are $O(3I_{3}L)$, $O(3I_{1}I_{3}L)$, and $O(3I_{2}I_{3}L)$, respectively. These values are less than the complexity of O(3ML) for the RCP algorithm. In summary, compared to the traditional algorithm, the proposed RCP-APH significantly reduces computational complexity when the number of iterations is large.

## 5. Simulation Analysis

In this section, a comprehensive simulation analysis was conducted to assess the performance of our algorithm. Each testing condition underwent 200 independent experiments and was accompanied by random noise and interference. Firstly, the rank estimation performance of the RCP-APH algorithm was compared with traditional information methods [9] and traditional RCP [30]. Secondly, under the assumption of accurate rank estimation, the parameter estimation performance of RCP-APH was compared with the performance of two mainstream tensor decompositions such as CP [12] and Tucker [10] as well as the RCP algorithm. Lastly, a detailed interference positioning performance comparison was conducted between the two variational methods.

According to the simulation conditions illustrated in Figure 1, the configuration is set as follows. The transmitting array is located at (0 m, 0 m), while the receiving array is positioned at (30 m, 0 m). The actual number of multipaths is 2, with the line-of-sight (LOS) path being obstructed. Simultaneously, the actual parameters for the dual-path channel are set as $\theta =[45^{\circ },30^{\circ }]$, $\phi =[45^{\circ },60^{\circ }]$, and τ=[142.13,136.60] ns. The signal bandwidth used is B=100 MHz, with Δτ·B=0.553. It is noted that the delay harmonic parameters are highly indistinguishable. Omnidirectional linear array antennas are equipped at both the transmitting and receiving ends and comprise $N_{BS-R}=N_{BS-T}=5$ antennas with spacing of $d_{t}=d_{r}=\lambda_{c}/2$. Under the above conditions, the uniqueness condition for CP decomposition is satisfied, as described in [14]. Moreover, the complex gains follow a circularly symmetric Gaussian distribution $\alpha_{l}∼CN\left(0,1/{\left(4\pi Df_{c}/c\right)}^{2}\;\right)$, where *c* is the speed of light, the LOS distance D=30 m, and the carrier frequency $f_{c}$ is 5.9 GHz. Considering the maximum aperture of the receiving array $A_{p}=\left(5-1\right)d_{r}$, we obtain $D\geq {2A_{p}^{2}/\lambda \;}_{c}=0.41$ m, satisfying the far-field assumption and belonging to the Fraunhofer zone for channel testing. Lastly and most importantly, CCI and FEI are taken into account in the simulation. Thus, at the tensor lattice level, we introduce the parameter of the interference ratio β, which describes the proportion of interference terms in the received tensor.

### 5.1. Initialization and Termination Conditions

For the variational methods, after performing variance normalization on the received tensor, we should also assume the Gaussian distribution of CN(0,I) for the factor matrices, which allows for the initialization of factor matrices without prior information. The initial rank $R_{int}$ is chosen as three times the number of true paths, i.e., six paths, satisfying the requirements of the weak upper bound, i.e., $R_{int}\leq \min_{n}\left(\sum_{i\neq n}I_{i}\right)$. In our model, the top-level hyperparameters, including $c_{0},d_{0},e_{0},f_{0},a_{0}^{\Xi },$ and $b_{0}^{\Xi }$, are set to 1 × $10^{-6}$, resulting in a noninformative prior. Thus, the expectation of hyperparameters can be initialized by $E\left[\Lambda \right]=I_{L}$, E[ν]=1, and $E[\gamma^{\Xi }]=1$. $V^{\left(n\right)}$ is simply set to $E[\Lambda^{-1}]$. For each category of interference, $E[S^{Tp}]$ is drawn from $CN(0,I_{Cst[Ind\left(Tp\right)]})$, while ${\left(\sigma^{Tp}\right)}^{2}$ is set to $E[{\left(\gamma^{Tp}\right)}^{-1}]$. The entire inference process of the model is summarized in Algorithm 1, where the posterior factors in Equation (9) are sequentially updated from bottom to top, as depicted in Figure 3. To enhance the speed of ARD for two variational algorithms in the presence of interference, redundant multipaths corresponding to ${\tilde{\lambda }}_{l}$ under the condition of E[λ]/min(E[λ])>η are eliminated. Additionally, for CP decomposition with known rank, the initial factor matrices are obtained using SVD operations. For the Tucker decomposition with known rank, we used the unitary ESPRIT algorithm with forward smoothing and HOSVD techniques.
**Algorithm 1** The proposed RCP-APH**Input:** a third-order complete received tensor Y, the IPTH of η, and the termination condition of $M_{Iters}$;
**Initialization:**  ${\tilde{A}}^{\left(n\right)}$, $V_{i_{n}}^{\left(n\right)}$, ∀n∈[1,N], $\forall i_{n}\in [1,I_{n}0,d_{0},e_{0},f_{0},a_{0}^{\Xi },b_{0}^{\Xi }$, $E[\gamma^{Tp}]=1$, ${\tilde{S}}^{Tp}∼CN\left(0,I_{Cst\left[Ind(Tp\right)]}\right)$, Tp∈Ξ=[CCI,FEI−R,FEI−T], the initial number of multipath is L, and Way=1 is used to indicate the dimension in which variational operations are in progress;
1: **while** 
$it\leq M_{Iters}$ 
**do**
2:      Tp=Ξ[Way]
3:      Increment variable it by 1;
4:      Increment variable Way by 1;
5:      **for** n=1 to *N* **do**
6:          Update the posterior $q_{n}\left(A^{\left(n\right)}\right)$ using (11);
7:      **end for**
8:      Update the posterior $q\left(\lambda \right)$ using (12);
9:      Update the posterior $q\left(\nu \right)$ using (15);
10:    Update the posterior $q\left(S^{Tp}\right)$ using (13);
11:    Update the posterior $q\left(\gamma^{Tp}\right)$ using (14);
12:    Evaluate the lower bound $ELBO^{Tp}\left(it\right)$ using (17);
13:    Reduce rank L by eliminating components of $E[\lambda ]/\min\left(E[\lambda ]\right)\;>\eta$;
14:    Ensuring alternating execution between dimensions Way=(Way==4)?1:Way;
15: **end while**
16: Calculate the channel parameters of $\left[\theta_{l},\phi_{l},\tau_{l},\alpha_{l}\right],\forall l\in \left[1,L\right]$.


### 5.2. Algorithm Performance

In the following, we choose the iteration number as $M_{Iters}=500$, the threshold of η=10, signal-to-noise ratio (SNR) ρ=20 dB, and number of subcarriers K=64. To control the iterations, we set β=0.2. Interference power is set as five times the noise power, i.e., $1/\gamma^{Tp}\;={\left(\sigma^{Tp}\right)}^{2}=5/\nu \;=5\sigma_{Noise}^{2}$. The CCI items ratio is 0.5. We consider narrowband interference, i.e., it appears at a limited number of consecutive frequency sampling (CFSs). In this standard setup, three CFSs are occupied by FEI, while two CFSs are occupied by CCI, as shown in Figure 2. The simulation results are depicted in Figure 4.

In Figure 4a, we primarily conduct a feasibility study on the proposed algorithm RCP-APH, where the interference power is calculated as the absolute power magnitude after variance normalization of the received tensor Y. The blue lines (solid, dotted, and dashed lines) depict the variations of ELBO for three different interferences, while the red solid line represents the estimated rank, i.e., the number of paths. The maximum number of iterations is 166, and at the 57th iteration, the algorithm achieves the true rank as indicated by the red dotted line. It is noteworthy that at the 57th iteration the ELBO unexpectedly decreases slightly, which can be explained by the fact that the redundant paths are eliminated, resulting in the loss of information entropy due to the small value of η. However, though choosing a large value of η may solve the problem of “unexpectedly decreases” in ELBO values, a large η value would also increase the number of iterations and, in turn, the complexity. Therefore, the threshold η must be selected appropriately in order to balance between the complexity and accuracy.

Figure 4b mainly analyzes the estimation performance of the RCP and RCP-APH algorithms. Firstly, the two curves in the figure represent the interference power distributions estimated by the two algorithms. It can be observed that, compared to the distribution estimated by the RCP-APH algorithm, the interference power estimated by the RCP shows a concentrated distribution, making it difficult to distinguish the true interference. Secondly, the solid and dashed vertical lines in the figure represent the noise power, signal power, and noise precision estimated by the both algorithms. The RCP-APH estimates the SNR more accurately compared to the RCP, as evidenced by the difference between the estimated signal power and noise power. Finally, in selecting the interference threshold, we consider the three aforementioned estimation metrics. If the estimated noise power is used as the threshold, the RCP would be unable to capture interference information. Therefore, this paper uses noise precision as the threshold for extracting interference terms. This threshold has the advantage of not only extracting the high-power interference estimated by the RCP but also facilitating subsequent performance comparisons of both algorithms.

### 5.3. Channel Estimation Performance

**Figure 5:**
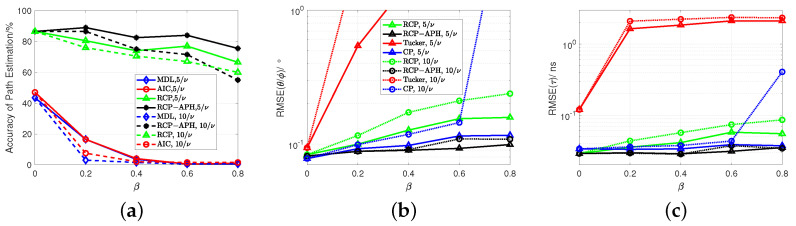
For different interference item ratios, a comparison of rank and parameter estimation performance is conducted for interference powers of $5\sigma_{Noise}^{2}$ and $10\sigma_{Noise}^{2}$. (**a**) Rank estimation. (**b**) Angle estimation. (**c**) Delay estimation. Here, (**b**,**c**) share a common legend.

Within this section, we evaluate the performance of rank estimation and channel parameter estimation. Firstly, a comparison of performance under different interference power ratios is conducted, as illustrated in Figure 5. In the rank estimation of Figure 5a, it is observed that information-theoretic methods, i.e., MDL and AIC, are ineffective in the presence of strong interference. This confirms the unsuitability of traditional information-theoretic approaches in the case of interference due to overfitting. As a result, channel parameter estimation algorithms based on matrix processing that strongly depend on rank estimation are significantly degraded. It is also evident that algorithms based on the variational model outperform information-theoretic methods.

Additionally, under low interference power (${\left(\sigma^{Tp}\right)}^{2}=5\sigma_{Noise}^{2}$), the RCP-APH algorithm surpasses RCP for all interference ratios. Under high interference power (${\left(\sigma^{Tp}\right)}^{2}=10\sigma_{Noise}^{2}$), RCP-APH only slightly lags behind RCP in the extremely unfavorable scenario of β=0.8. This reveals the robustness of the proposed algorithm.

In Figure 5b,c, it can be observed that the proposed RCP-APH outperforms other algorithms and reveals its robustness against changes in interference power as indicated by the black line. Furthermore, traditional RCP exhibits a certain degree of robustness. However, due to the lack of actual interference modeling, its performance is comparatively inferior, as indicated by the green lines. Moreover, for methods that require the number of multipaths to be known, such as the CP and Tucker decomposition methods, CP shows better performance due to its effective reduction of interference in single dimensions through multidimensional iterations. On the other hand, Tucker decomposition, due to HOSVD, encompasses interference information from multiple dimensions, resulting in the poorest performance.

### 5.4. Interference Estimation Performance

**Figure 6:**
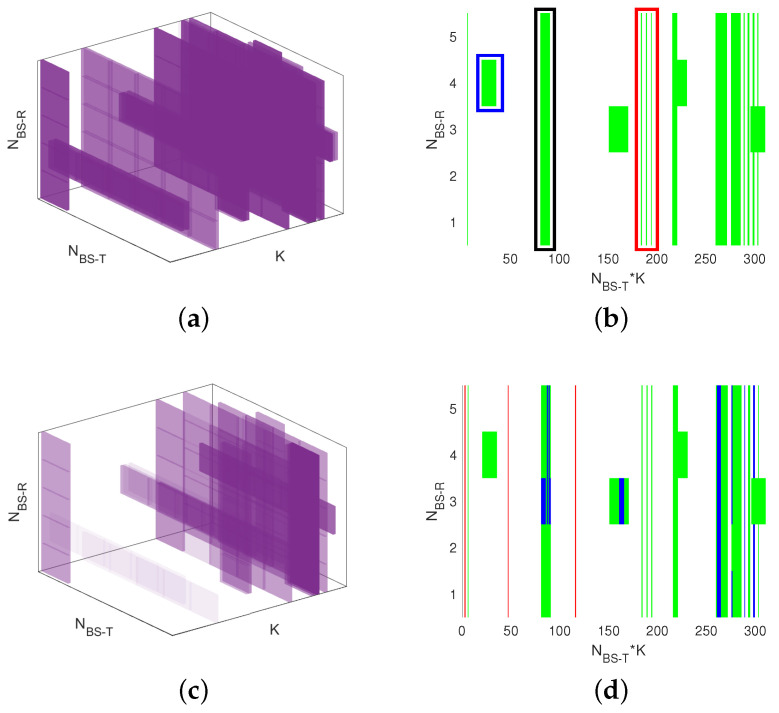
Study on the performance of interference estimation for the RCP-APH. Green indicates accurately estimated interference positions, while blue represents unestimated interference positions. (**a**) True interference; (**b**) matrix unfolding of true interference; (**c**) estimated interference; (**d**) matrix unfolding of estimated interference.

A comparison is conducted in terms of the performance of time–frequency position estimation for interference. In this context, “time” represents the large-scale sampling time, denoted as *t*, not to be confused with the small-scale delay τ. The term “frequency” denotes the position of frequency sampling points for interference. Since this paper processes all sub-channel snapshots at a single sampling time *t*, the discussion is thereby simplified to identifying the interference position at frequency sampling points. Here, a simulation of the RCP-APH algorithm is performed under conditions of β=0.2 and ρ=20 dB. The received tensor is illustrated in Figure 2, and the interference parameter estimation is depicted in Figure 6.

Figure 6b depicts the unfolding form of Figure 6a along the 1-mode pattern. The vertical axis has a size of $N_{BS-R}$, and the horizontal axis has a size of $N_{BS-T}\cdot K$. The green color in Figure 6b denotes the specific positions of the interference in the channel tensor. The red box visualizes an FEI-T that is composed of three vertical green lines, indicating that all receiving antennas are affected by interference from the same transmitter antenna for three CFSs. The blue box shows an FEI-R that occupies $N_{BS-T}$ units in the horizontal direction and that lasts for three CFSs. Further, the black block represents a CCI that spans over both the vertical and horizontal directions with two CFSs. In Figure 6c, the RCP-APH accomplishes interference estimation for a single realization. It is evident in Figure 6a that the lower-power regions, indicated by lighter colors, cannot be identified due to their power approaching the noise level. In Figure 6d, which is the unfolding of Figure 6c, interference items underestimated by the algorithm are represented in blue. Notably, the majority of interferences are accurately estimated, as indicated by the green color.

**Figure 7:**
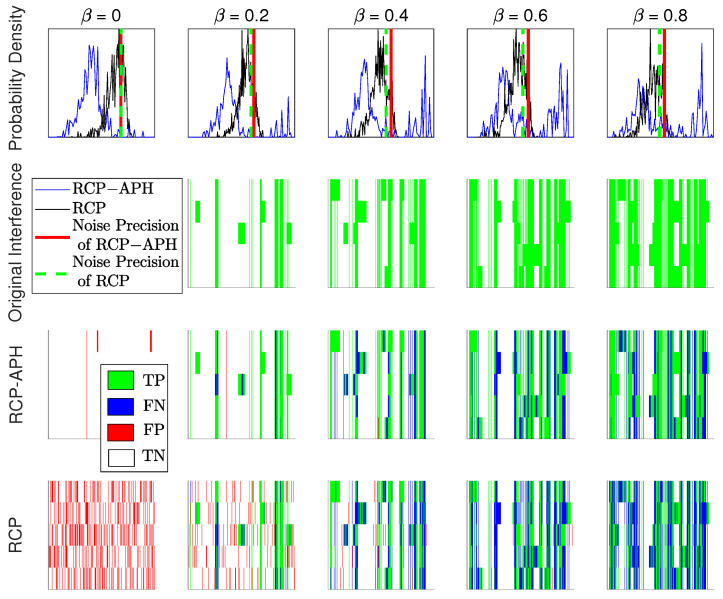
The estimations of the interference positions are compared between two variational algorithms under different interference ratios. To clearly depict the performance differences between the algorithms, coordinate annotations for all subplots are omitted. The first row illustrates the estimated noise precision and PDF of the interference item power for both the RCP-APH and RCP algorithms. The coordinate scales are consistent with Figure 4b. The second row represents the actual interference, while the third and fourth rows depict the estimations of the interference positions for both algorithms. The coordinate scales align with those in Figure 6b.

In Figure 7, a single experimental comparison of two variational algorithms is conducted under different interference ratios. To better demonstrate the difference in performance, we use the same coordinate systems as in Figure 4b and Figure 6b. These plots in the first row depict the PDF of the interference power at different values of β. The second row represents the specific positions, where the true interference is in the unfolded form. The third and fourth rows show the estimation of interference positions for both the RCP-APH and RCP algorithms. Here, the performance metric for position estimation based on the binary classification model in [33] is adopted. True positives (TPs) indicate accurately identified interference positions as depicted in green; false negatives (FNs) represent missed detections of interference positions, shown in blue; false positives (FPs) denote incorrectly identified interference positions as shown in red; and true negatives (TNs) signify correctly identified positions without interference, depicted in white. Seen from Figure 7, the proposed RCP-APH algorithm can distinguish interference by an optimal threshold of noise precision. As evident in the subsequent three rows of the figure, both algorithms exhibit a decreasing trend in red and an increasing trend in blue with the rise of β. This corresponds to the actual mapping: transitioning from overestimation to underestimation. The distinct advantages of the proposed algorithm include: 1. There are rare occurrences of singular interference item estimation, enabling direct mapping between interference items and actual interference. 2. The proposed algorithm can discern the actual interference ratio, while the traditional RCP fails under β=0.

The statistical characteristics of the interference in the 200 independent experiments maintain the same conditions as in Section 5.3. The subsequent analysis employs three binary performance parameters as follows: 1. $Precision=\mathrm{TP}/\left(\mathrm{TP}+\mathrm{FP}\right)$ is utilized to depict the accuracy of estimations; 2. $Recall=\mathrm{TP}/\left(\mathrm{TP}+\mathrm{FN}\right)$ signifies how many of the actual estimations are captured; 3. $F1\;Score=2\cdot Precision\cdot Recall/\left(Precision+Recall\right)$ provides a comprehensive balance between the first two metrics.

**Figure 8:**
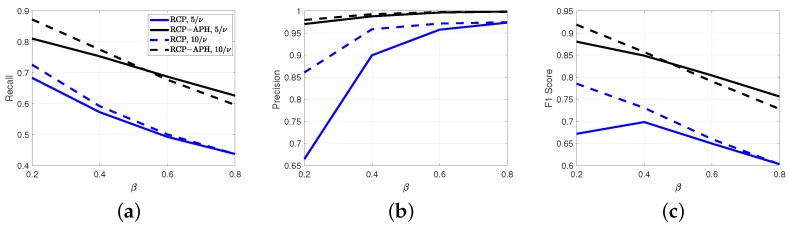
Under different interference item ratios, a comparison of interference estimation is conducted for interference powers of $5\sigma_{Noise}^{2}$ and $10\sigma_{Noise}^{2}$. (**a**) Recall. (**b**) Precision. (**c**) F1 Score. Here, all subplots share a common legend.

As illustrated in Figure 8, the three performance metrics of the RCP algorithm increase with the growth of interference power. However, the performance gains associated with the interference power gradually diminish as β increases. A notable distinction between the RCP-APH and the RCP is that for β≥0.6, there is a decline in recall, leading to a corresponding decrease in the F1 score. Importantly, the most crucial point is that across various interference powers, all three performance metrics of the proposed algorithm consistently surpass those of the RCP algorithm by a significant margin.

**Figure 9:**
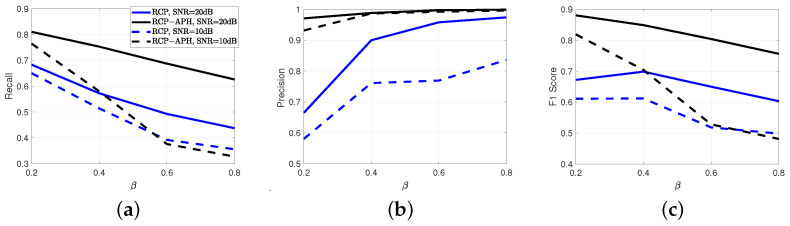
Interference estimation performance is compared for different interference item ratios for both 10 dB and 20 dB of ρ. (**a**) Recall. (**b**) Precision. (**c**) F1 Score. Here, all subplots share a common legend.

In Figure 9, it is evident that as ρ decreases to 10 dB, all three performance metrics of both methods decline. The proposed algorithm exhibits slightly inferior performance compared to the RCP under low-ρ and high-β conditions. However, under high-ρ conditions and low-ρ with low-β conditions, RCP-APH demonstrates superior performance.

**Figure 10:**
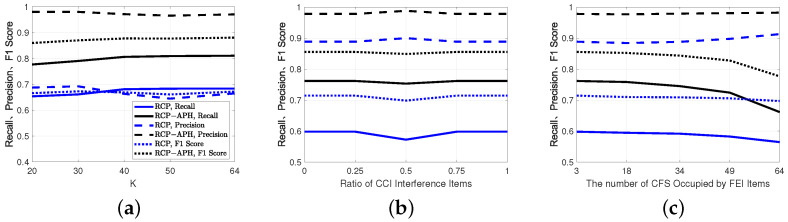
Performance metrics for interference estimation for different spatial structures and interference characteristics. (**a**) Different sampling K. (**b**) Different ratio of CCI. (**c**) Different bandwidth of FEI. All subplots share a common legend.

From Figure 10a, it can be observed that with the increase in frequency points *K*, there is a slight decrease in precision for RCP-APH, while recall and F1 score exhibit a monotonic increase, significantly outperforming the RCP. As shown in Figure 10b, the three performance metrics remain nearly constant. However, due to incomplete observations, this outlier appears when the interference ratio reaches 50%. According to Figure 10c, widening in the interference bandwidth results in an improvement in precision for both algorithms, while recall and F1 score decline. Importantly, the estimation performance of RCP-APH consistently outperforms that of RCP.

## 6. Conclusions

In this paper, we propose a robust RCP based on the APH to interference. With the strong correlation of the interference, the proposed algorithm is capable of simultaneous estimation of the rank, channel, and interference parameters. In comparison with the RCP, the proposed algorithm has the following features: 1. Increasing the model sparsity reduces the computational complexity. 2. The noise precision, from which interference items can be inferred, is reasonably and accurately estimated. 3. The estimated interference items show spatial correlation, enabling more accurate identification of the type of interference. 4. The prior hypothesis aligns more closely with real interference, enhancing the overall performance of communication systems. Through a simulation analysis, a comprehensive examination was conducted using different SNRs, interference powers, tensor spatial structures, proportions of interference items occupied by CCI, and lengths of the interference bandwidth. This analysis provides conclusive evidence of the superior estimation performance of rank and channel parameters using the RCP-APH algorithm. Finally, the accurate interference time–frequency position estimation performance of the proposed algorithm is validated.

## Data Availability

The data are contained within the article.

## Appendix A

$$\begin{array}{cc} \; & L\left(q,Tp\right)=E_{q(\Theta )}\left[\ln p\left(Y-{\tilde{S}}^{\backslash Tp},\Theta \right)\right]+H\left(q\left(\Theta \right)\right)\\ \; & =E_{q\left(\left\{A^{\left(n\right)}\right\},S^{Tp},\nu \right)}\left[\ln p\left(Y-{\tilde{S}}^{\backslash Tp}|\left\{A^{\left(n\right)}\right\},S^{Tp},\nu^{-1}\right)\right]+E_{q\left(\left\{A^{\left(n\right)}\right\},\lambda \right)}\left[\sum_{n=1}^{3}\ln p\left(A^{\left(n\right)}|\lambda \right)\right]\\ \; & +E_{q\left(S^{Tp},\gamma^{Tp}\right)}\left[\ln p\left(S^{Tp}|\gamma^{Tp}\right)\right]+E_{q(\lambda )}\left[\ln p\left(\lambda \right)\right]+E_{q(\gamma^{Tp})}\left[\ln p\left(\gamma^{Tp}\right)\right]+E_{q(\nu )}\left[\ln p\left(\nu \right)\right]\\ \; & -E_{q\left(\left\{A^{\left(n\right)}\right\}\right)}\left[\sum_{n=1}^{3}\ln q\left(A^{\left(n\right)}\right)\right]-E_{q\left(S^{Tp}\right)}\left[\ln q\left(S^{Tp}\right)\right]-E_{q(\lambda )}\left[\ln q\left(\lambda \right)\right]\\ \; & -E_{q(\gamma^{Tp})}\left[\ln q\left(\gamma^{Tp}\right)\right]-E_{q(\nu )}\left[\ln q\left(\nu \right)\right], \end{array}$$

where

$$\begin{array}{cc} \; & E_{q\left(\left\{A^{\left(n\right)}\right\},S^{Tp},\nu \right)}\left[\ln p\left(Y-{\tilde{S}}^{\backslash Tp}|\left\{A^{\left(n\right)}\right\},S^{Tp},\nu^{-1}\right)\right]\\ \; & =-\prod_{n}I_{n}\ln\left(\pi \right)+\prod_{n}I_{n}E_{q}\left[\ln\nu \right]-E_{q}\left[\nu \right]E_{q}\left[{∥Y-{\tilde{S}}^{\backslash Tp}-\left[\;\left[A^{\left(1\right)},A^{\left(2\right)},A^{\left(3\right)}\right]\;\right]-S^{Tp}∥}_{F}^{2}\right]\\ \; & =-\prod_{n}I_{n}\ln\left(\pi \right)+\prod_{n}I_{n}\left[\psi \left(e_{M}\right)-\ln f_{M}\right]-\frac{e_{M}}{f_{M}}E_{q}\left[{∥Y-{\tilde{S}}^{\backslash Tp}-\left[\;\left[A^{\left(1\right)},A^{\left(2\right)},A^{\left(3\right)}\right]\;\right]-S^{Tp}∥}_{F}^{2}\right], \end{array}$$

$$\begin{array}{cc} \; & E_{q\left(\left\{A^{\left(n\right)}\right\},\lambda \right)}\left[\sum_{n=1}^{3}\ln p\left(A^{\left(n\right)}|\lambda \right)\right]=E_{q}\left[\sum_{n}\sum_{i_{n}}\left\{-L\ln\pi +\ln|\Lambda |-a_{i_{n}}^{(n)H}\Lambda a_{i_{n}}^{\left(n\right)}\right\}\right]\\ \; & =\sum_{n}\left\{-LI_{n}\ln\pi +I_{n}\sum_{l}E_{q}\left[\ln\lambda_{l}\right]-\sum_{i_{n}}E_{q}\left[a_{i_{n}}^{(n)H}\Lambda a_{i_{n}}^{\left(n\right)}\right]\right\}\\ \; & =-L\sum_{n}I_{n}\ln\pi +\sum_{n}I_{n}\sum_{l}E_{q}\left[\ln\lambda_{l}\right]-\sum_{n}\sum_{i_{n}}\left\{\sum_{l}E_{q}\left[a_{i_{n},l}^{(n)*}a_{i_{n},l}^{\left(n\right)}\right]E_{q}\left[\ln\lambda_{l}\right]\right\}\\ \; & =-L\sum_{n}I_{n}\ln\pi +\sum_{n}I_{n}\sum_{l}\left(\psi \left(c_{M}^{l}\right)-\ln d_{M}^{l}\right)-\mathrm{Tr}\left\{\tilde{\Lambda }\sum_{n}\left({\tilde{A}}^{(n)H}{\tilde{A}}^{\left(n\right)}+I_{n}V^{\left(n\right)}\right)\right\}, \end{array}$$

$$\begin{array}{cc} \; & E_{q\left(S^{Tp},\gamma^{Tp}\right)}\left[\ln p\left(S^{Tp}|\gamma^{Tp}\right)\right]\\ \; & =E_{q}\left[\sum_{Ind(Tp)}Cst\left(\backslash Ind(Tp)\right)\left(\ln\gamma_{Ind(Tp)}^{Tp}-\ln\pi \right)-\gamma_{Ind(Tp)}^{Tp}\sum_{\backslash Ind(Tp)}{\left|S_{i_{1},i_{2},i_{3}}^{Tp}\right|}^{2}\right]\\ \; & =-\prod_{n}I_{n}\ln\pi +Cst\left(\backslash Ind(Tp)\right)\sum_{Ind(Tp)}\left(\psi \left(a_{M}^{\gamma_{Ind(Tp)}}\right)-\ln b_{M}^{\gamma_{Ind(Tp)}}\right)\\ \; & -\sum_{Ind(Tp)}\left(\sum_{\backslash Ind(Tp)}\frac{a_{M}^{\gamma_{Ind(Tp)}}}{b_{M}^{\gamma_{Ind(Tp)}}}\left({\left(\sigma_{i_{1},i_{2},i_{3}}^{TP}\right)}^{2}+{\left|{\tilde{S}}_{i_{1},i_{2},i_{3}}^{Tp}\right|}^{2}\right)\right), \end{array}$$

$$E_{q}\left[\ln p\left(\lambda \right)\right]\;=-L\ln\Gamma \left(c_{0}^{l}\right)+Lc_{0}^{l}\ln d_{0}^{l}+\sum_{l}\left\{\left(c_{0}^{l}-1\right)\left(\psi \left(c_{M}^{l}\right)-\ln d_{M}^{l}\right)-d_{0}^{l}\frac{c_{M}^{l}}{d_{M}^{l}}\right\},$$

$$\begin{array}{cc} \; & E_{q}\left[\ln p\left(\gamma^{Tp}\right)\right]=-Cst\left(Ind(Tp)\right)\ln\Gamma \left(a_{0}^{Tp}\right)+Cst\left(Ind(Tp)\right)a_{0}^{Tp}\ln b_{0}^{Tp}\\ \; & \;\;\;\;\;\;+\sum_{Ind(Tp)}\left\{\left(a_{0}^{Tp}-1\right)\left(\psi \left(a_{M}^{\gamma_{Ind(Tp)}}\right)-\ln b_{M}^{\gamma_{Ind(Tp)}}\right)-b_{0}^{Tp}\frac{a_{M}^{\gamma_{Ind(Tp)}}}{b_{M}^{\gamma_{Ind(Tp)}}}\right\}, \end{array}$$

$$E_{q}\left[\ln p\left(\nu \right)\right]\;=-\ln\Gamma \left(e_{0}\right)+e_{0}\ln f_{0}+\left(e_{0}-1\right)\left(\psi \left(e_{M}\right)-\ln f_{M}\right)-f_{0}\frac{e_{M}}{f_{M}}\;,$$

$$-E_{q}\left[\sum_{n=1}^{3}\ln q\left(A^{\left(n\right)}\right)\right]=\sum_{n}I_{n}L\left(\ln\pi +1\right)+\sum_{n}I_{n}\left|V^{\left(n\right)}\right|,$$

$$-E_{q}\left[\ln q\left(S_{\Omega }^{Tp}\right)\right]=\prod_{n}I_{n}\left(1+\ln\pi \right)+\sum_{\Omega }\ln{\left(\sigma_{\Omega }^{Tp}\right)}^{2},$$

$$-E_{q}\left[\ln q\left(\lambda \right)\right]=\sum_{l=1}^{L}\left\{\ln\Gamma \left(c_{M}^{l}\right)-\left(c_{M}^{l}-1\right)\psi \left(c_{M}^{l}\right)-\ln d_{M}^{l}+c_{M}^{l}\right\},$$

$$-E_{q}\left[\ln q\left(\gamma^{Tp}\right)\right]=\sum_{Ind(Tp)}\left\{\ln\Gamma \left(a_{M}^{\gamma_{Ind(Tp)}}\right)-\left(a_{M}^{\gamma_{Ind(Tp)}}-1\right)\psi \left(a_{M}^{\gamma_{Ind(Tp)}}\right)-\ln b_{M}^{\gamma_{Ind(Tp)}}+a_{M}^{\gamma_{Ind(Tp)}}\right\},$$

$$-E_{q}\left[\ln q\left(\nu \right)\right]=\ln\Gamma \left(e_{M}\right)-\left(e_{M}-1\right)\psi \left(e_{M}\right)-\ln f_{M}+e_{M}\;.$$
